# Occurrence of Intersex in the Marine Mussel *Perumytilus purpuratus* (Mollusca: Bivalvia): Does Gonadal Parasitism Play a Role?

**DOI:** 10.3390/biology14010070

**Published:** 2025-01-14

**Authors:** Pablo A. Oyarzún, Sebastián Diaz, Sara M. Rodríguez, Gonzalo Ruiz-Tagle, José J. Nuñez, Jorge E. Toro

**Affiliations:** 1 Centro de Investigación Marina Quintay (CIMARQ), Universidad Andres Bello, Quintay 2340000, Chile; sebastiandiazsepulveda@gmail.com (S.D.); gjruiztagle@uc.cl (G.R.-T.); 2 Departamento de Ecología, Facultad de Ciencias, Universidad Católica de la Santísima Concepción, Concepción 4030000, Chile; saramrodriz@gmail.com; 3 Centro de Investigación en Recursos Naturales y Sustentabilidad (CIRENYS), Universidad Bernardo O’Higgins, Avenida Viel 1497, Santiago de Chile 8370993, Chile; 4 Instituto de Ciencias Marinas y Limnológicas, Universidad Austral de Chile, Independencia 631, Valdivia 5090000, Chile; jjnunezn@gmail.com (J.J.N.); jorgetoroyagui@gmail.com (J.E.T.)

**Keywords:** dioecy, gonadal tissue, intertidal, mollusks, parasites, sexual systems

## Abstract

Intersexuality is a reproductive phenomenon that occurs in some gonochoric species and refers to the simultaneous presence of both male and female gametes within the same individual. Although this phenomenon has been reported in various invertebrate species, many aspects remain poorly understood, especially in marine mussels. However, it has been suggested that the prevalence of parasites within populations could induce the occurrence of intersex animals. We studied intersexuality in the marine mollusk *Perumytilus purpuratus*, a key species of the rocky intertidal zone on the southeastern coast of the Pacific Ocean. A total of 6472 mussels from eight locations in northern and southern Chile were analyzed. We estimated the size of the specimens, the sex ratio of the population, and the prevalence of parasites. Additionally, we examined the germ cells of intersex mussels. The results showed that the male-to-female sex ratio (1:1) was maintained in the populations. Intersex mussels were found in six of the eight locations, representing 0.19% of the mussels analyzed. However, no parasites were found in the intersex animals. Additionally, the abundance of intersex individuals was not correlated with parasitism levels in the population. In intersex mussels, the gonadal tissue was compartmentalized, with male and female germ cells remaining separate. It is concluded that intersexuality in *Perumytilus purpuratus* is a low-frequency reproductive phenomenon, likely resulting from an alteration in the sex determination mechanism. Intersexuality offers a valuable opportunity to explore the biological aspects of sex determination in mussels. Therefore, further research in this area should be pursued.

## Simple Summary

Intersexuality is a rare biological phenomenon where an individual has both male and female reproductive cells, despite belonging to a species typically characterized by separate sexes. While it has been observed in some bivalves, its causes and effects on populations remain poorly understood. This study focused on the marine mollusk *Perumytilus purpuratus*, a key species inhabiting the rocky coasts of the southeastern Pacific. Mussels from eight locations in Chile were analyzed to determine how common intersexuality is and how it manifests within populations. Intersex mussels were identified in six of the eight sites, with a low frequency within each population. The gonads of these intersex mussels displayed distinct compartments for male and female reproductive cells. The frequency of intersexuality was not linked to parasite levels, which had been hypothesized as a potential cause. This study concludes that intersexuality in *P. purpuratus* is a rare occurrence, likely resulting from alterations in the mechanisms of sex determination. These findings enhance our understanding of sex differentiation in mussels and provide valuable insights for broader studies on reproductive biology.

## 1. Introduction

Intersexuality is a reproductive phenomenon that occurs in some dioecious species, characterized by the simultaneous presence of both male and female gametes within an individual. In other words, signs of hermaphroditism appear in one or more populations of gonochoric species [1]. Intersexuality has been documented across several species, with crustaceans and mollusks being particularly prevalent [2]. In mollusks, studies have predominantly focused on gastropods [1]. Regarding the causes, various factors have been described as contributors to intersexuality in natural populations. For example, environmental pollution has been identified as a cause, as certain chemical compounds can induce feminization or masculinization in animals (see Langston et al. [3]). In mollusks, the occurrence of intersexuality has been linked to exposure to endocrine-disrupting compounds (EDCs) in the environment [4,5]. However, intersex animals have been found in areas without contaminants, suggesting that intersexuality may be influenced by other factors. The ecological implications of intersexuality may be evolutionarily significant. High levels of intersexuality may impact population dynamics and reproductive success, potentially leading to reduced fitness in affected individuals [6]. These aspects remain poorly understood in mollusks.

Parasitism has been identified as a factor associated with intersexuality, linked to both the feminization of males and the masculinization of females [2,7,8,9,10]. For example, Ohtsuka et al. [11] observed that intersexuality in the mysid *Siriella japonica* was associated with parasitism, as intersex individuals were found to harbor parasites. In these cases, the feminization of males appeared to result from parasite-induced castration. Such dynamics could have broader implications; if parasitism disproportionately affects one sex within a population, it may lead to a skewed sex ratio, potentially reducing overall fecundity. Despite these observations, the effects of parasitism on the reproductive success of marine invertebrates remain insufficiently studied [12].

The family Mytilidae includes a variety of bivalve species, but research has yet to report conclusive findings of intersex in this family. The marine mussel *Perumytilus purpuratus* (Lamarck, 1819), commonly known as the “chorito maico”, is a benthic invertebrate inhabiting rocky intertidal zones [13]. It is distributed from Ecuador to the Strait of Magellan in the Pacific Ocean and extends along the Argentine Atlantic coast to Santa Cruz [14]. This species plays an important ecological role by providing refuge for various species. It achieves this by forming three-dimensional matrices that can host a wide variety of marine organisms [15,16].

*Perumytilus purpuratus* is a gonochoric species with external fertilization. Reproduction occurs primarily during the spring and summer, with less intense reproductive activity taking place in the winter [17]. Additionally, *P. purpuratus* serves as an intermediate host for three species of Platyhelminthes (Digenea): (i) *Prosorhynchoides carvajali* from the family Bucephalidae [18,19], (ii) *Proctoeces sicyases* [20], and (iii) an unidentified trematode species belonging to the family Fellodistomidae [19,21]. The parasitic infection occurs during the mussels’ filtration process. Once inside, the parasites localize in the gonadal tissue, leading to the effective castration of the affected individuals [18,21]. High parasite loads can negatively impact fertilization rates and potentially disrupt the sex ratio within mussel populations.

In this context, we hypothesize that if parasites are capable of inducing castration in *P. purpuratus* and if the parasitic load in a population is high, then such infection could disrupt the sex ratio, potentially leading to a reproductive imbalance. This imbalance can result in the emergence of intersex individuals as a compensatory reproductive response.

The objective of this study was to investigate the role of parasitism in the occurrence of intersexuality in the intertidal mussel *Perumytilus purpuratus* along approximately 1120 km of the coastline. Additionally, this study aimed to elucidate the reproductive characteristics of intersex individuals by analyzing their germ cells to better understand this sexual phenotype.

## 2. Materials and Methods

### 2.1. Mussel Collection

**Figure 1:**
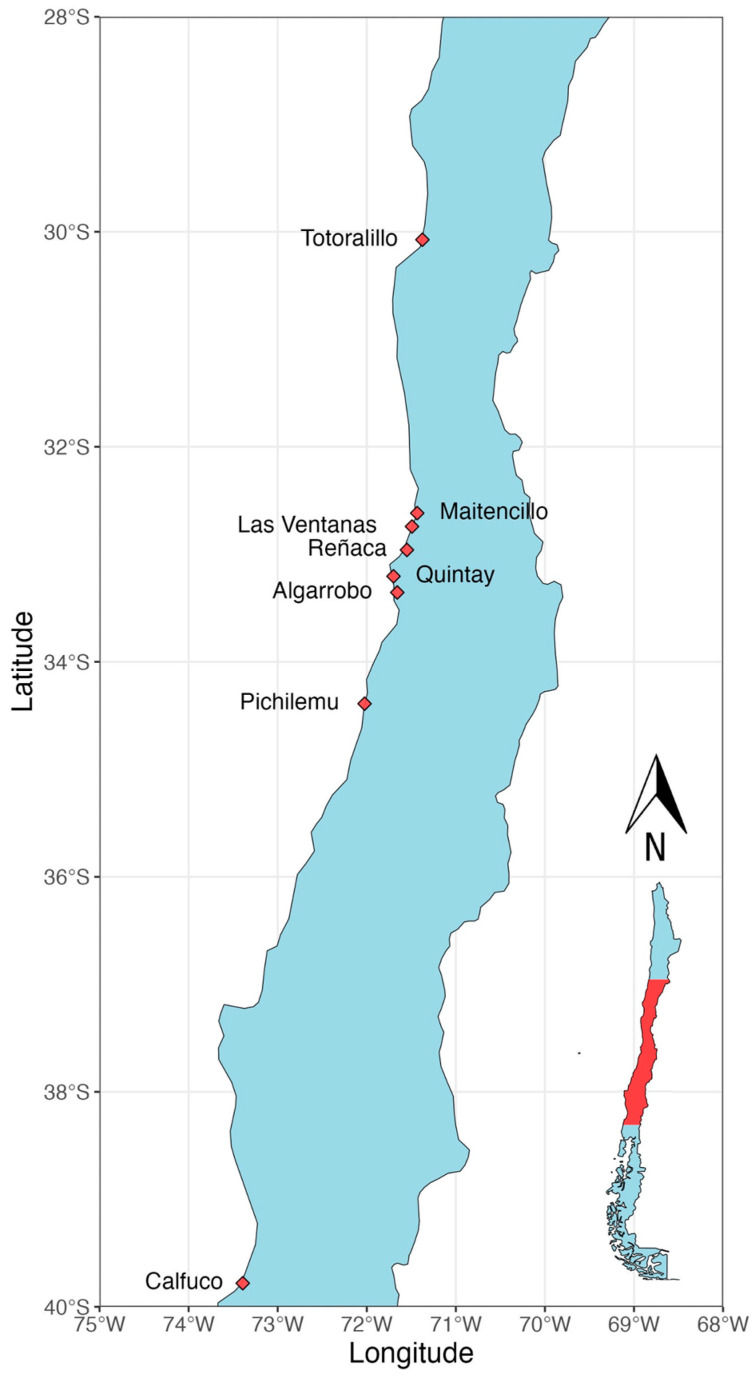
Map of the study region along the Chilean coast, showing *Perumytilus purpuratus* sampling sites in rocky intertidal zones.

A total of 6472 adult mussels were collected from January 2023 to August 2023 from eight locations (see Table 1 and Figure 1). These locations are separated by approximately 1120 km and exhibit contrasting patterns of environmental variability. Animals from the lower, middle, and upper intertidal zones were sampled. Live mussels were placed in labeled plastic bags and transported at a low temperature (~4 °C) to the Invertebrate Reproductive Biology Laboratory for dissection.

### 2.2. Sex Identification in Mussels

Each mussel’s maximum length was measured using a digital caliper. Subsequently, the sex of each animal was determined based on macroscopic and microscopic observations following the criteria reported by Oyarzún et al. [17,22]. The female tissue in the females exhibited a brown coloration, contrasting with the characteristic whitish color of the males. Additionally, to confirm the sex, a gonad smear was prepared, and germ cells were examined under a microscope (Olympus Corporation, Tokyo, Japan CX21, 40X).

### 2.3. Analysis of Gonadal Parasitism

Since the parasite infection occurs solely in the reproductive tissue of mature mussels, only adult individuals were analyzed. The procedure involved examining the gonadal tissue under a stereoscopic microscope, and the parasite morphology was analyzed following the descriptions previously reported by Muñoz et al. [19,23].

### 2.4. Histological Procedure and Gametogenic Analysis

Germ cells present in the gonadal tissue of unisexed and intersex mussels were analyzed. For this, adult mussels from Reñaca and Pichilemu (Table 1) of both sexual types were collected. To ensure sample integrity, the animals were kept alive until the moment of dissection. A scalpel was used to extract the gonad from each specimen, which was subsequently fixed in Davidson’s solution for 48 h. The histological procedure (tissue dehydration and embedding) followed the methodology described by Oyarzún et al. [17]. Longitudinal sections of the gonadal lobe, with a thickness of 7 μm, were cut from each sample. The histological sections were stained with hematoxylin and eosin [24].

From the histological samples, the gamete volume fraction (GVF), representing the proportion of germinal tissue in the gonads, was estimated. The methodology described by Toro et al. [25] was employed for this purpose. Digital photographs of each histological section (male/female tissue of unisexed and intersex individuals) were captured using an Olympus SC180 camera and subsequently analyzed with ImageJ v1.53a software [26]. Data were obtained by calculating the percentage of germinal tissue (follicles) and interfollicular (or connective) tissue. Additionally, the percentage of previtellogenic, vitellogenic, mature, and atresic oocytes in the female tissue of unisexed and intersex mussels was estimated using the methodology described by Oyarzún et al. [17,22].

### 2.5. Statistical Analysis

To determine whether the sex ratio in *Perumytilus purpuratus* deviates significantly from the expected 1:1 ratio, a repeated G–test of goodness-of-fit (*p* < 0.05) was employed, as described by Sokal & Rohlf [27]. This test assesses the nominal variables of sex (males and females) and locality, aiming to identify any deviations from expected proportions and to evaluate whether there is significant variation across different localities.

A factorial ANOVA was conducted to compare the sizes of male, female, and intersex mussels, with sex (female, male, intersex) and location (eight sites) as the factors. Prior to performing the ANOVA, the assumptions of homogeneity of variance and normality were tested using Levene’s test and the Kolmogorov–Smirnov test, respectively. When significant differences were detected, post hoc comparisons were carried out using the Tukey test to identify specific group differences.

To examine the occurrence of intersexuality in *Perumytilus purpuratus*, a 3 × 2 contingency table was initially constructed to evaluate the frequencies of intersex individuals across different intertidal zones—classified as low, mid, and high—at various sampled localities. Given the low frequency and sparse distribution of intersex individuals across these zones and localities, Fisher’s exact test for count data was applied to assess the potential associations between these variables. Furthermore, to address the issue of data sparsity, Firth’s bias-reduced logistic regression was employed using the ‘logistf’ function within the “logistf” R package. This regression model aimed to investigate the impact of intertidal zones and parasite prevalence on the log-odds of intersex occurrence, providing a robust analysis of the extreme rarity of intersex individuals. This model is particularly suitable for situations where the event of interest is extremely rare, as it addresses issues of complete or quasi-complete separation and corrects the bias associated with maximum likelihood estimates in small sample sizes.

The frequencies of parasitized mussels across different intertidal zones were analyzed using a 3 × 2 contingency table, and the independence of these variables was assessed with a chi-squared test. In cases where statistically significant dependencies were detected, post hoc pairwise comparisons were performed with Bonferroni correction for multiple testing. This approach aimed to identify specific intertidal zone combinations that exhibited significant associations with parasitism.

All analyses were performed using R v.3.3.3 [28].

## 3. Results

### 3.1. Sex Ratio and Intersex in Perumytilus purpuratus

**Figure 2:**
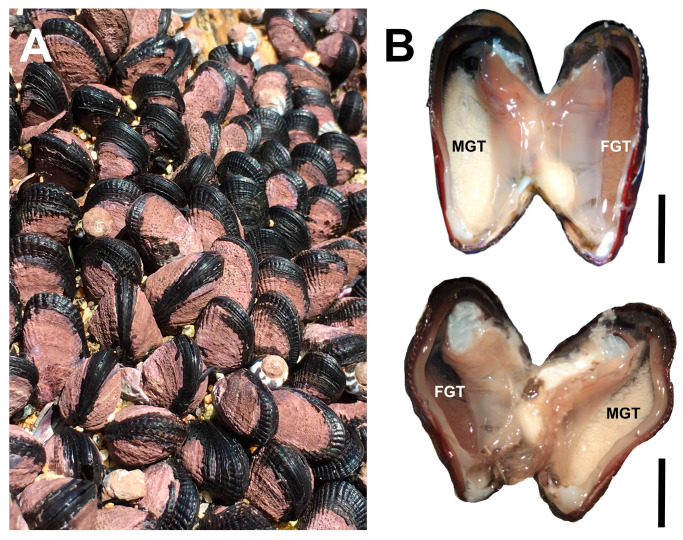
Photograph of a *Perumytilus purpuratus* bed in the intertidal zone, Chile (**A**), and examples of the location of reproductive tissue in intersex mussels (**B**). The female gonad is indicated by brown tissue (FGT: female gonadal tissue), while the male gonad is indicated by yellow tissue (MGT: male gonadal tissue). The bar corresponds to 1 cm.

A total of 6472 mussels were examined, of which 50.62% were female, 49.19% were male, and 0.19% were intersex (see intersex mussel—Figure 2). The results of the repeated G-test of goodness-of-fit indicated no significant differences in the sex ratio from the expected 1:1 ratio. The observed values for males and females did not deviate significantly from the expected equal distribution. The test statistic was G = 11.74 with a *p*-value of 0.16, suggesting that the null hypothesis of a 1:1 sex ratio could not be rejected (Table S1).

**Figure 3:**
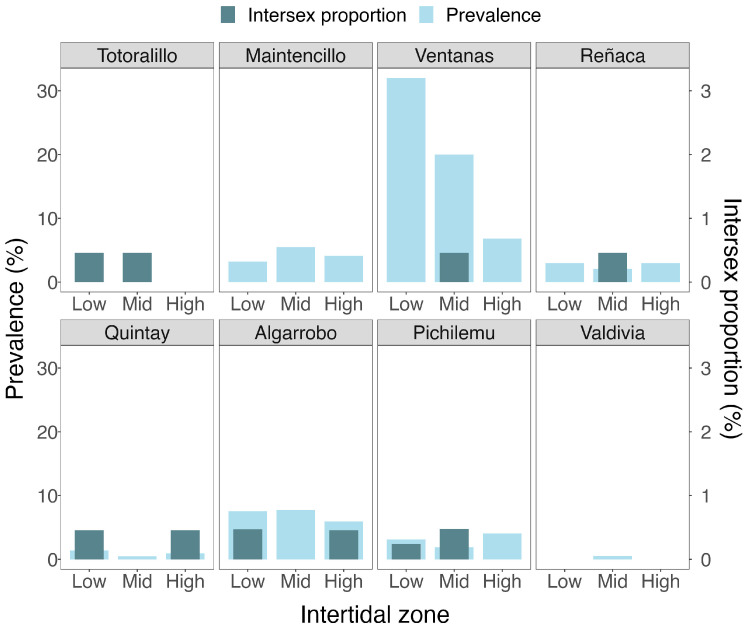
Proportion of intersex individuals and prevalence of parasites in *Perumytilus purpuratus* beds in the lower, middle, and high intertidal zones of the analyzed localities in Chile.

Intersex mussels were found in six out of the eight locations, representing between 0.15% and 0.32% of the analyzed specimens (Figure 3). Despite the presence of intersex individuals, Fisher’s exact test for count data showed no significant association between intersexuality and the different intertidal zones (*p* > 0.05; Table S2). This suggests that the occurrence of intersex individuals was not significantly influenced by intertidal zone distribution.

### 3.2. Size of Intersex Mussels

The lengths of the analyzed animals ranged from 10.4 to 38.4 mm, while the intersex mussels had lengths ranging from 19.2 to 32.3 mm. No significant differences were found among the sizes of males, females, and intersex specimens (F_2,6150_ = 0.828; *p* > 0.44—Table S3).

### 3.3. Gonadal Parasitism

In the gonadal tissue of *Perumytilus purpuratus*, parasites from the Fellodistomidae and Bucephalidae families (*Prosorhynchoides carvajali*) were found. The overall parasite prevalence was 4.37%. Although *P. carvajali* had the highest prevalence among the analyzed mussels (3.91%), in the Quintay locality, the most abundant parasite belonged to the Fellodistomidae family. The highest prevalence of parasites in the gonads of the mussels was recorded in the localities of Las Ventanas (19.55%) and Algarrobo (7.06%), which did not show a high occurrence of intersex (Figure 3). Furthermore, no germ cells were observed in the host mussels.

More parasites were found in mussels located in the middle and lower intertidal zones (76% of the parasites). Pearson’s chi-squared test indicated a significant dependency between the occurrence of parasitized mussels and intertidal zones (*p* < 0.05; Table S4), suggesting that the distribution of parasitized mussels is not independent of the intertidal zone. Additionally, post hoc pairwise comparisons with Bonferroni *p*-value adjustment revealed a significant difference in the prevalence of parasitized mussels between the high and low zones (*p* < 0.001; Table S5). However, no significant differences were observed between the low and mid zones, nor between the mid and high zones, indicating that the high and low intertidal zones differ significantly in terms of parasitized mussel prevalence, while the mid zone does not differ significantly from either the low or high zones.

Firth’s bias-reduced logistic regression results showed that neither intertidal zones nor the occurrence of parasitized mussels significantly impacted the prevalence of intersex individuals (*p* > 0.05 for all predictors—see Figure 3; Table 2).

### 3.4. Gametogenesis of Intersex Mussels

**Figure 4:**
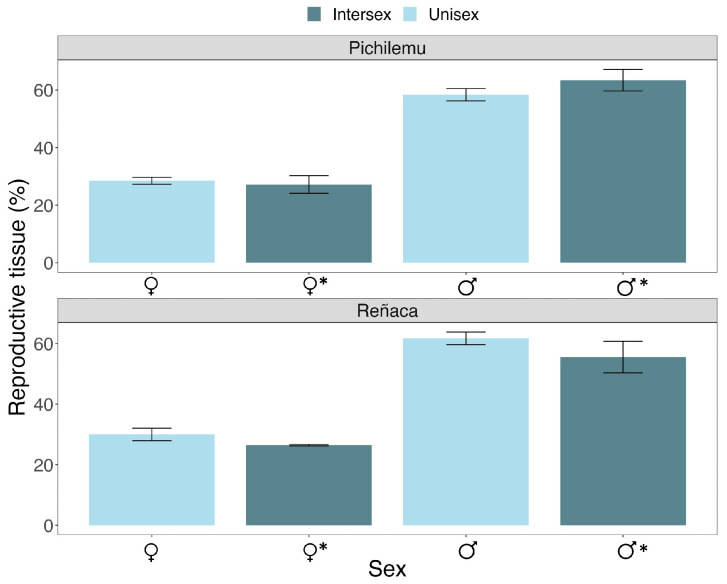
Percentage of reproductive tissue (GVF) in the gonads of gonochoric (male and female) and intersex individuals of the bivalve *Perumytilus purpuratus* from Reñaca and Pichilemu, Chile. The asterisk represents the tissue of intersex mussels.

**Figure 5:**
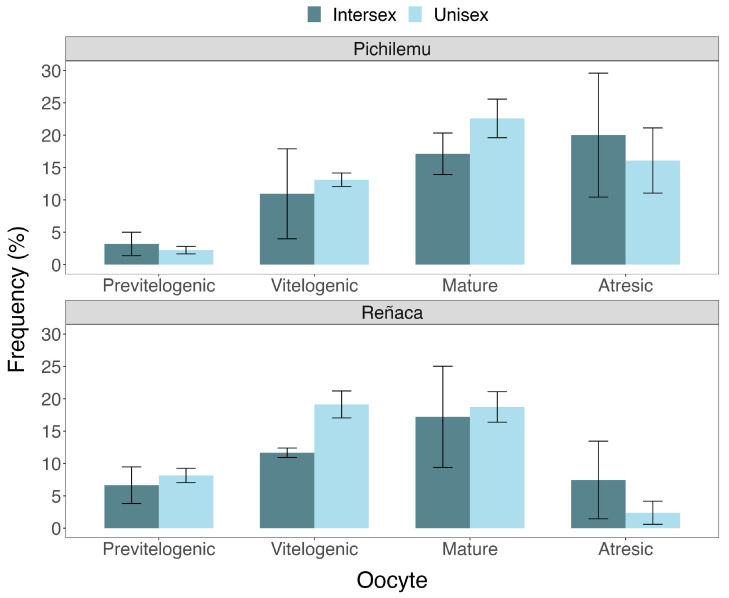
Percentage of oocyte stages (previtellogenic, vitellogenic, mature, atresic) within the gonadal tissue of gonochoric and intersex females of *Perumytilus purpuratus* from Reñaca and Pichilemu, Chile.

In intersex animals, the most common gonadal arrangement was the presence of male tissue in the right valve and female tissue in the left valve (58.33%). There were no significant differences in the amount of germinal tissue when comparing the male and female gonads of intersex and gonochoric mussels (♂_intersex_ = 61% ± 5 and ♂_unisex_ = 60% ± 4; ♀_intersex_ = 29 ± 3 and ♀_unisex_ = 31 ± 2) (Figure 4). Additionally, oocyte analysis revealed that both gonochoric and intersex females exhibited all four types of oocyte cells during the gametogenic maturity period in Reñaca and Pichilemu, highlighting the presence of vitellogenic and mature oocytes (Figure 5). However, atresic oocytes were more abundant in Pichilemu (♀_intersex_ = 20% ± 8; ♀_unisex_ = 16% ± 3), indicating gametogenic differences between the mussels from the two locations (Figure 5).

## 4. Discussion

### 4.1. Sex Ratio in Perumytilus purpuratus

The sex ratio in *Perumytilus purpuratus* aligns with the expected 1:1 ratio of males to females. This finding suggests the existence of a biological mechanism that regulates and maintains a balanced number of individuals of each sex within populations. Several studies have reported that the sex ratio is a critical factor for species stability (e.g., Ancona et al. [29]). This is supported by the theory of sex allocation, which refers to how organisms allocate their reproductive resources between the production of males and females. The ability to maintain this balance indicates a sophisticated biological control with a genetic basis. Therefore, the sex ratio is a fundamental parameter in population dynamics.

Our results show that intersexuality in *Perumytilus purpuratus* is a low-frequency reproductive phenomenon that is not associated with the intertidal zone where the mussels inhabit, indicating no environmental effect on the occurrence of intersexual individuals. Reports in the literature suggest that intersexuality can arise from disruptions in sex determination in species with an environmental sex determination (ESD) system. Biotic or abiotic factors, such as food quality or temperature variations, have been identified as triggers, along with certain contaminants [5,30,31,32]. However, our findings indicate that this is not the case for *P. purpuratus*, as intersexuality was assessed in locations with varying environmental conditions, including temperature, salinity, and food availability [33]. Moreover, Las Ventanas Beach, a site with high levels of heavy metal contamination due to decades of industrial activity [34], did not show the highest incidence of intersexuality. This suggests that a historically contaminated beach did not exhibit an increased number of intersex mussels.

Overall, our results align with findings from other geographical areas, where intersexuality was not linked to any environmental variable (e.g., *Mytilus* spp.—Dublinowska et al. [35]). However, studies quantifying the proportion of intersex individuals in mussels are scarce; most have focused on reporting their occurrence, and sometimes these cases have been erroneously categorized as hermaphroditism. Although intersexuality exhibits phenotypic characteristics similar to hermaphroditism, it is not a reproductive strategy adopted by the species. A misclassification complicates the verification of cases and the determination of a normal range for the occurrence of intersexuality, distorting the data and underestimating its true prevalence in animals.

### 4.2. Parasitism and Intersex

Unlike what was reported in mysids by Ohtsuka et al. [11], our study found no intersex mussels with parasites, thereby ruling out the possibility that parasites induce sexual modification in *Perumytilus purpuratus*. Additionally, no germ cells were observed in the host mussels, suggesting a process of castration in unisexual individuals. Parasite infection was only observed in the reproductive tissue of mature mussels, as previously described by Muñoz et al. [23]. In contrast to other reports (e.g., Short et al. [9,10]), the mussel parasites do not have the ability to feminize or masculinize their hosts. Parasites identified in the gonadal tissue of these mussels belonged to the families Fellodistomidae and Bucephalidae, with a total prevalence of 4.37%. However, the highest prevalences were recorded in the localities of Las Ventanas (19.55%) and Algarrobo (7.06%), where intersexuality was relatively low. Moreover, there was a correlation between parasitized mussels and the intertidal zones they inhabited, with more parasites found in mussels closer to the water. This reflects the life cycle of the parasites, which is associated with the presence of other marine hosts.

Parasites reproduce sexually in the intestine of the clingfish *Syciaces sanguineus*. Female parasites produce eggs that are released into the water through the host’s feces, where they hatch into ciliated larvae called “miracidia”. These larvae enter mussels through filtration. Once inside, the parasites attach to the gonad tissue, where they reproduce asexually, forming sporocysts. Subsequently, cercariae are released into the water and find their second intermediate host, limpets of the species *Fissurella* spp., attaching to their intestines and developing into a third larval stage called metacercaria. Finally, the cycle is completed when clingfish prey on the limpets [21,36,37,38,39,40]. Therefore, our results suggest that the location of mussels in the intertidal zone may influence the reproductive biology of *P. purpuratus* due to gonadal castration by parasites, but without inducing intersexuality in the mussels.

### 4.3. Reproductive Potential of Intersex Mussels

In *P. purpuratus*, intersexuality exhibited a compartmentalization of male and female gonadal tissue within individuals, without any lateral preference for the location of the gonadal tissue. This could be a strategy to achieve optimal follicular development by preventing cellular competition between male and female germ cells.

Gametogenic analysis revealed no significant differences in the amount of germinal tissue between the male and female gonads of intersex and unisexual (gonochoric) mussels. This finding suggests that the gametogenic potential of intersexual mussels is comparable to that of unisexual mussels, implying that the acquisition and use of energy for reproduction in intersex mussels may be similar to that in male and female mussels in the population. Additionally, atresic oocytes were observed in the female tissue of both groups, indicating a reallocation of germinal energy as a strategy to conserve reproductive energy at the end of the gonadal cycle [41]. Furthermore, the gametogenic differences observed between the various localities are attributed to environmental factors such as food availability and temperature. These variables directly affect the development of germ cells in marine mussels [17]. Therefore, although the development of gametes in intersexual mussels may follow a course similar to that of gonochoric mussels, there are local variations associated with the location where they live.

## 5. Conclusions

The intersexuality of *Perumytilus purpuratus* is a reproductive phenomenon of low frequency, with no apparent pattern among the analyzed localities and intertidal zonation. Parasitism also showed no relation to its occurrence. This suggests that it may be a biological event caused by some alteration in sex determination. Therefore, the intersexuality of *Perumytilus purpuratus* offers an opportunity to study the underlying biological aspects related to male and female determination in populations. These aspects are poorly understood within Mollusca [42], which is why the intersexuality model of *Perumytilus purpuratus* offers a valuable opportunity to search for candidate genes involved in sex determination (see Breton et al. [43]). However, further research is needed to explore intersexuality in species of the Mytilidae family to broaden our understanding of this reproductive phenomenon in natural populations.

## Figures and Tables

**Table 1:** Site survey information, number of specimens collected or processed (*N*), date of collection, and geographical coordinates of sites.

Site	Coordinates	*N*	Date
Totoralillo	30°04′25.8″ S; 71°22′30.2″ W	660	20 February 2023
Maitencillo	32°37′01.9″ S; 71°26′00.9″ W	660	27 June 2023
Las Ventanas	32°44′29.0″ S; 71°29′32.2″ W	660	23 January 2023
Reñaca	32°57′33.9″ S; 71°32′56.0″ W	1320	04 September 2023
Quintay	33°12′16.3″ S; 71°41′59.2″ W	660	31 January 2023
Algarrobo	33°21′18.2″ S; 71°39′27.2″ W	627	4 January 2023
Pichilemu	34°23′23.1″ S; 72°01′27.6″ W	1260	13 September 2023
Calfuco	39°46′51.7″ S; 73°23′31.7″ W	600	20 March 2023
Total		6472	

**Table 2:** Firth’s bias-reduced logistic regression results for intersex occurrence.

Predictors	Coefficient	Std Error	Lower 95% CI	Upper 95% CI	X^2^	*p* Value
Low zone	0.617	0.777	−0.891	2.364	0.637	0.425
Mid zone	0.968	0.735	−0.396	2.663	1.888	0.169
Parasited	−0.184	1.396	−5.038	1.851	0.017	0.896

## Data Availability

All data generated or analyzed during this study are included in the published article (and its Supplementary Information Supplementary Files).

## Supplementary Materials

The following supporting information can be downloaded at https://www.mdpi.com/article/10.3390/biology14010070/s1, Table S1: Repeated G-test of goodness of fit, testing sex ratio along coastal sites in Chile; Table S2: Contingency table for intersex mussel occurrence across intertidal zones, assessed using Fisher’s exact test; Table S3: Three-way ANOVA of total length differences by sex, site, and intertidal zone, including interaction effects; Table S4: Contingency tables for parasitized mussel occurrence pooled across intertidal zones, assessed using Pearson’s Chi-squared test; Table S5: Pairwise comparisons of proportions with Bonferroni P adjustment for parasitized mussel occurrence between intertidal zones level combinations with respect to intertidal high zone.
