# Supplementary material for: Occurrence of Intersex in the Marine Mussel Perumytilus purpuratus (Mollusca: Bivalvia): Does Gonadal Parasitism Play a Role?
Source: Biology (Basel). 2025 Jan 14;14(1):70. doi: 10.3390/biology14010070 (PMC11762098; doi:10.3390/biology14010070)
Supplement: Supplementary file 1 [file biology-14-00070-s001.zip › biology-3386525-supplementary.pdf]

Table S1. Repeated G-test of goodness of fit, testing sex ratio along coastal sites in Chile.

Site	Sex			G-value	df	*P* value
	F	M				
Totoralillo	321	337		0.389	1	0.533
Maitencillo	314	324		0.157	1	0.692
Las Ventanas	277	253		1.087	1	0.297
Reñaca	634	645		0.095	1	0.758
Quintay	337	315		0.742	1	0.389
Algarrobo	317	290		1.201	1	0.273
Pichilemu	662	575		6.124	1	0.113
Calfuco	281	315		1.941	1	0.164
			total G	11.736	8	0.163
Pooled	3143	3054	pooled G	1.278	1	0.258
			heterogeneity G	10.458	7	0.164

Table S2. Contingency table for intersex mussel occurrence across intertidal zones, assessed using Fisher's Exact Test.

Intertidal zone	Mussel number	
	Gonochoric	Intersex
Low	2030	4
Mid	2066	6
High	2101	2

Fisher's Exact Test for Count Data: *P*-value = 0.3416

Table S3. Three-way ANOVA of total length differences by sex, site, intertidal zone, including interaction effects.

**Source**	**df**	**Sum square**	**Mean square**	**F**	***P* value**
Sex	2	29	14.7	0.828	0.44
Site	7	11135	1590.8	89.297	< 2e-16
Zone	2	232	115.9	6.506	0.002
Sex : Site	12	92	7.6	0.429	0.95
Sex : Zone	4	65	16.3	0.916	0.45
Site: Zone	14	17455	1246.8	69.990	< 2e-16
Sex : Site: Zone	16	182	11.4	0.640	0.85
Residuals	6150	109558	17.8	-	-

Table S4: Contingency tables for parasitized mussel occurrence pooled across intertidal zones, assessed using Pearson's Chi-squared test.

	**Mussel number**	
**Intertidal zone**	**Non-parasited**	**Parasitized**
Low	2029	122
Mid	2067	92
High	2091	69

$X^2 = 15.888$, df = 2, P-value = 0.0003548

Table S5. Pairwise comparisons of proportions with Bonferroni *P* adjustment for parasitized mussel occurrence between intertidal zones level combinations with respect to intertidal high zone.

**Intertidal zone**	**High**	**Low**
Low	0.00032	-
Mid	0.23014	0.1178
